# Profiling the Effects of Systemic Antibiotics for Acne, Including the Narrow-Spectrum Antibiotic Sarecycline, on the Human Gut Microbiota

**DOI:** 10.3389/fmicb.2022.901911

**Published:** 2022-05-31

**Authors:** Ines B. Moura, Ayman Grada, William Spittal, Emma Clark, Duncan Ewin, James Altringham, Emilio Fumero, Mark H. Wilcox, Anthony M. Buckley

**Affiliations:** ^1^Healthcare-Associated Infections Group, Leeds Institute of Medical Research, Faculty of Medicine and Health, University of Leeds, Leeds, United Kingdom; ^2^Almirall LLC, Malvern, PA, United States; ^3^Department of Microbiology, Leeds Teaching Hospital NHS Trust, Old Medical School, Leeds General Infirmary, Leeds, United Kingdom; ^4^Microbiome and Nutritional Science Group, School of Food Science, Faculty of Food Science and Nutrition, University of Leeds, Leeds, United Kingdom

**Keywords:** sarecycline, acne (acne vulgaris), long-term antibiotic treatment, gut microbiota, *in vitro* model, minocycline, doxycycline

## Abstract

Treatment for moderate-to-severe acne vulgaris relies on prolonged use of oral tetracycline-class antibiotics; however, these broad-spectrum antibiotics are often associated with off-target effects and negative gastrointestinal sequelae. Sarecycline is a narrow-spectrum antibiotic treatment option. Here, we investigated the effect of prolonged sarecycline exposure, compared with broad-spectrum tetracyclines (doxycycline and minocycline) upon the colonic microbiota. Three *in vitro* models of the human colon were instilled with either minocycline, doxycycline or sarecycline, and we measured microbiota abundance and diversity changes during and after antibiotic exposure. Significant reductions in microbial diversity were observed following minocycline and doxycycline exposure, which failed to recover post antibiotic withdrawal. Specifically, minocycline caused a ~10% decline in Lactobacillaceae and Bifidobacteriaceae abundances, while doxycycline caused a ~7% decline in Lactobacillaceae and Bacteroidaceae abundances. Both minocycline and doxycycline were associated with a large expansion (>10%) of Enterobacteriaceae. Sarecycline caused a slight decline in bacterial diversity at the start of treatment, but abundances of most families remained stable during treatment. Ruminococcaceae and Desulfovibrionaceae decreased 9% and 4%, respectively, and a transient increased in Enterobacteriaceae abundance was observed during sarecycline administration. All populations recovered to pre-antibiotic levels after sarecycline exposure. Overall, sarecycline had minimal and transient impact on the gut microbiota composition and diversity, when compared to minocycline and doxycycline.

## Introduction

Acne vulgaris (acne) is a chronic disease characterized by inflammatory lesions of the skin with different degrees of severity. Acne has been reported as one of the 10 most common diseases globally (Hay et al., 2014), with Europe, North America and South America showing the highest burden (Karimkhani et al., 2017; Laughter et al., 2020). However, acne has an estimated prevalence of 9.4% of the worldwide population when all age groups are considered (Vos et al., 2012).

Treatment for moderate-to-severe acne often involves prescription of tetracycline-class antibiotics for long-term use, such as minocycline or doxycycline (Armstrong et al., 2020). These are broad-spectrum antibiotics with activity against Gram-positive and Gram-negative bacteria. Although all antibiotics can impact the gut microbiota, the effect is detrimental with broad-spectrum antibiotics (Dethlefsen et al., 2008; Chilton et al., 2015; Francino, 2016; Elvers et al., 2020). Side effects resulting from prolonged broad-spectrum antibiotic exposure are likely to be associated with the prolonged or permanent disruption of the commensal populations that constitute the normal gut microbiota, which is known as antibiotic-mediated dysbiosis. The use of minocycline in acne patients was found to be associated with significant dysbiosis of the skin and the gut microbiota (Armstrong et al., 2020; Baldwin, 2020; Thompson et al., 2020). Although no causal link has been established, the use of doxycycline in acne patients was found to be associated with a 2.25-fold increased risk of developing Crohn’s disease, an inflammatory bowel disease (IBD; Graber, 2021). Sarecycline is a narrow-spectrum third generation tetracycline derivative, recently approved by the US Food and Drug Administration for the treatment of moderate to severe acne vulgaris (Moore et al., 2019). Sarecycline is particularly active against the Gram-positive anaerobe, *Cutibacterium acnes* (formerly *Propionibacterium acnes*), and also aerobic Gram-positive cocci, such as *Staphylococcus aureus* (including MRSA), but has reduced *in vitro* activity against other aerobic and anaerobic bacteria, such as *Klebsiella pneumoniae*, *Escherichia coli*, *Bifidobacterium bifidum*, and *Lactobacillus acidophilus* (Zhanel et al., 2018). For example, the MIC_50_ of sarecycline, doxycycline and minocycline against *E. coli* was 16, 2 and 1 mg/L, respectively. Similarly, sarecycline is less active *in vitro* compared with doxycycline and minocycline against both enteric Gram-positive and Gram-negative anaerobes. This profile for sarecycline is due to its narrow spectrum of activity, which is associated with low rates of gastrointestinal adverse events seen in clinical trials (Armstrong et al., 2020).

Antibiotic therapy is an essential tool for acne treatment, but studies focusing on longitudinal samples to elucidate the effects of prolonged use of tetracycline-class drugs in the human gut bacteria are still lacking. A study investigating fecal samples from eight patients before and after 4 weeks of treatment with minocycline reported a depletion in several species of *Lactobacillus* and *Bifidobacterium* but no significant decrease in microbial diversity. However, no samples were collected during treatment (Thompson et al., 2020).

Our team has previously used an *in vitro* model of the human colon to successfully investigate population changes of microbial species during antibiotic instillation, to profile the outgrowth of *Clostridioides difficile* as a result of antimicrobial therapy, and to test therapeutic interventions to resolve *C. difficile* infection (Freeman et al., 2003; Moura et al., 2019; Buckley et al., 2020; Roberts et al., 2020). This system has been validated against the intestinal contents of sudden death victims and shown to accurately reproduce the *in vivo* physicochemical and microbiological measurements.

In this study, three independent gut models were used to assess the long-term effects of either sarecycline, minocycline, and doxycycline exposure on the human gut microbiota and the recovery of the microbiota after withdrawal of the antibiotics.

## Materials and Methods

### The *in vitro* “Gut Model”

**Figure 1:**
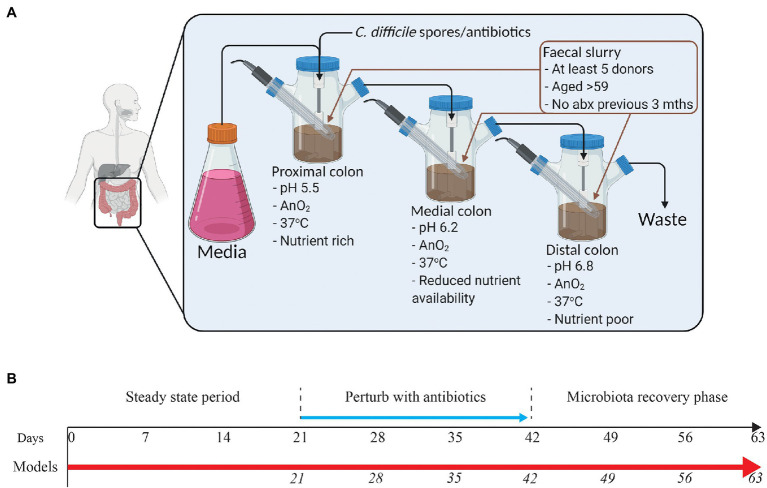
Schematic representation of the human *in vitro* triple chemostat “gut model” **(A)** and timeline of the experimental design **(B)**. Following initialization with a pooled fecal slurry, sarecycline (model S; 17 mg/L once daily), minocycline (model M; 19.3 mg/L twice daily), or doxycycline (model D; 22 mg/L once daily) were administered into vessel 1 for 21 days in each of the three models (blue arrow). Microbial populations were monitored daily throughout the experimental timeline. Days in italics show when samples for DNA extraction were **(A)** was created using BioRender.

The model consists of a triple-stage system with vessels arranged in a weir cascade and top-fed with a complex growth medium at a controlled rate (*D* = 0.015 h^−1^) as previously described (Moura et al., 2019). Each vessel represents the proximal colon (vessel 1, pH 5.5 ± 0.2), medial colon (vessel 2, pH 6.2 ± 0.2), or the distal colon (vessel 3, pH 6.8 ± 0.2), reflecting the *in vivo* physiological conditions in pH, oxygen content, temperature, transit time, and nutrient availability (Figure 1A). All vessels are continuously stirred, and anaerobically maintained at 37°C.

At the start of the experiment, each vessel was seeded with a fecal slurry (10% w/v) made from fecal donations from five healthy adults with no history of antibiotic use in the previous 3 months.

### Experimental Design

A total of three gut models were set as described above and run in these experiments. For each model, the microbial populations were allowed to stabilize for 2 weeks until reaching a “steady state.” Each model received a different antibiotic at a concentration reflective of the reported antibiotic colonic values in humans. Antibiotics were instilled as follows: Model S was dosed with sarecycline (16.99 mg/L once daily for 21 days; Ayman Grada, personal communication from unpublished clinical trial data), model M was dosed with minocycline (19.3 mg/L twice daily for 21 days; Steigbigel et al., 1968; Macdonald et al., 1973), and model D was dosed with doxycycline (22 mg/L once daily for 21 days; Macdonald et al., 1973; Nord and Heimdahl, 1986). Microbiota recovery was monitored for 3-weeks post antibiotic (Figure 1B), as this period allows stability of drug concentration to be achieved in the gut model. As such, we were able to access the maximum impact of the gut levels of the drug on the microbiota.

### Bacteria Enumeration Using Direct Culture

Bacterial populations were sampled daily in each of the three models, in which a 10-fold dilution series of this sample was plated out onto various selective and non-selective agars for enumeration as described previously (Buckley et al., 2021). Bacterial colonies were enumerated and confirmed using microbiological and biochemical techniques. The limit of detection using this method is <1.2 log_10_ CFU/ml. Comparative analysis of key bacterial populations used this enumeration data from the day prior to antibiotic instillation as baseline data, to calculate the fold change in bacterial enumeration from baseline to the day of last antibiotic instillation, or the last day of recovery (3-weeks post-antibiotic instillation). A bacterial population was considered to be significantly impacted when the log_2_ −2 ≤ fold change ≥2 in comparison with baseline enumerated levels.

### Antibiotic Bioassays

The concentration of each antibiotic was determined by bioassays as previously described (Buckley et al., 2021). Briefly, to determine the concentration of sarecycline and minocycline, indicator organism *Kocuria rhizophila* (ATCC 9341) was inoculated into Wilkins-Chalgren agar plates. A calibration series of the antibiotic was added to each plate alongside the samples. Zone diameters were measured, and concentration curves plotted so that unknown concentrations from vessel supernatants could be determined. Assays were performed in triplicate. Concentrations of doxycycline were determined as described above but using *S. aureus* (ATCC 29213) as indicator microorganism and iso-sensitest agar as growth media.

### DNA Extraction and 16S rRNA Sequencing

All three models were sampled for bacterial 16S testing at key timepoints. Sampling regimen is shown in Figure 1B. Four 1-ml aliquots from vessel 3 of each model were collected, spun down (14,000 rpm for 20 min at 4°C), and the pellet used for DNA extraction using FastDNA^TM^ Spin Kit for Soil (MPBio, United Kingdom), following manufacturer’s instructions. Extracts were stored at −80°C.

Bacterial 16S rRNA V4 region libraries were prepared as previously detailed (Buckley et al., 2021). Sequencing was performed on a MiSeq (Illumina) with 250 bp paired end reads.

### Taxonomic Analysis and Diversity Analysis

Demultiplexed FASTQ files of 16S sequences were trimmed of adapter sequences using CLC Genomics Workbench (version 20.0.4). The paired reads were joined together and assembled into the contigs, and quality controlled based on the parameters such as maxambig = 0, minlength = 150 and maxlength = 500 (default settings). Unique sequences were aligned against the SILVA reference database (v132 97%). OTUs (operational taxonomic units) were identified by clustering the sequences and were assigned the consensus taxonomy information with label = 0.03. For the diversity measurements, Shannon diversity index was computed for all samples and the distance matrix based on 0.5 UniFrac distance was used for principal coordinates (PCoA) analysis and visualization for each group of samples.

### Data Access

Datasets related to this article can be found hosted at Sequence Read Archive under BioProject ID: PRJNA744500.

## Results

### Recapturing the Human Microbiota in the Gut Model

Three independent *in vitro* gut models, primed with a pooled fecal slurry, were left untreated to allow the microbial populations to equilibrate (Figure 1A). Comparison of the microbial populations between the fecal slurry and each model (model S, model M, and model D) showed a high level of similarity and bacterial family compositional abundance between the recaptured microbiota in each model and the fecal slurry used to seed each vessel (Supplementary Figure S1). Once the microbiota established, we instilled either sarecycline (model S), minocycline (model M), or doxycycline (model D) at reported concentrations found within the human colon, daily for 3 weeks (Figure 1B), and assessed microbial kinetics during antibiotic exposure and following antibiotic withdrawal.

### Effect of Sarecycline on the Microbiota

**Figure 2:**
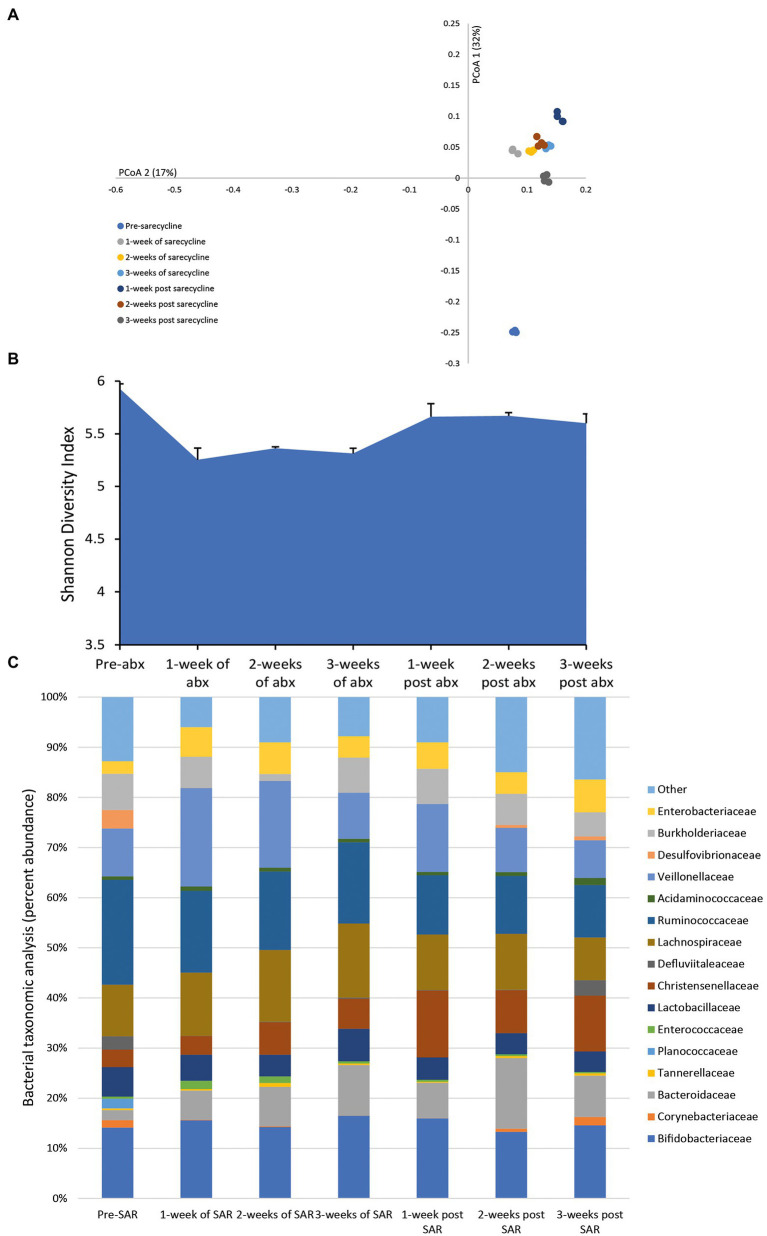
Effect of sarecycline on the human microbiota. Changes to the microbiota profile during each week of sarecycline instillation and the recovery phase shown as a Principal Coordinate Analysis plot **(A)**. Results shown are four technical replicates. Changes in the microbial diversity after exposure to sarecycline and the recovery phase, as measured by the Shannon diversity index from four technical replicates **(B)**. Bacterial taxonomic abundance changes in response to sarecycline instillation **(C)**. Stacked bar graphs are mean percent abundance, from four technical replicates, analyzed from 16S rRNA sequencing.

Once daily dosing of sarecycline over 3 weeks achieved a mean bioactive concentration of 15.9 mg/L (range over 3 weeks dosing period, 14.28–19.86 mg/L) in the gut model; however, the level of sarecycline was undetectable 3 days after cessation of dosing. After the first week of antibiotic dosing, the microbial patterns showed an initial minor decline in the Shannon diversity index, an indicator of sample diversity (Figures 2A,B). However, subsequent administration of sarecycline for 2 additional weeks did not result in further changes to the microbial diversity or composition, and diversity increased after antibiotic withdrawal. The abundancies of many of the bacterial families remained unchanged following exposure to sarecycline; however, during the first week of sarecycline instillation, an initial decline in the abundance of Ruminococcaceae (↓ 9.1%) and Desulfovibrionaceae (↓ 3.7%) was observed, which recovered to pre-sarecycline levels before antibiotic instillation had finished (Figure 2C). This was mirrored by a modest increase to the Enterobacteriaceae (↑3.4%) abundance, which decreased to pre-antibiotic levels by the end of sarecycline instillation.

### Effect of Minocycline on the Microbiota

**Figure 3:**
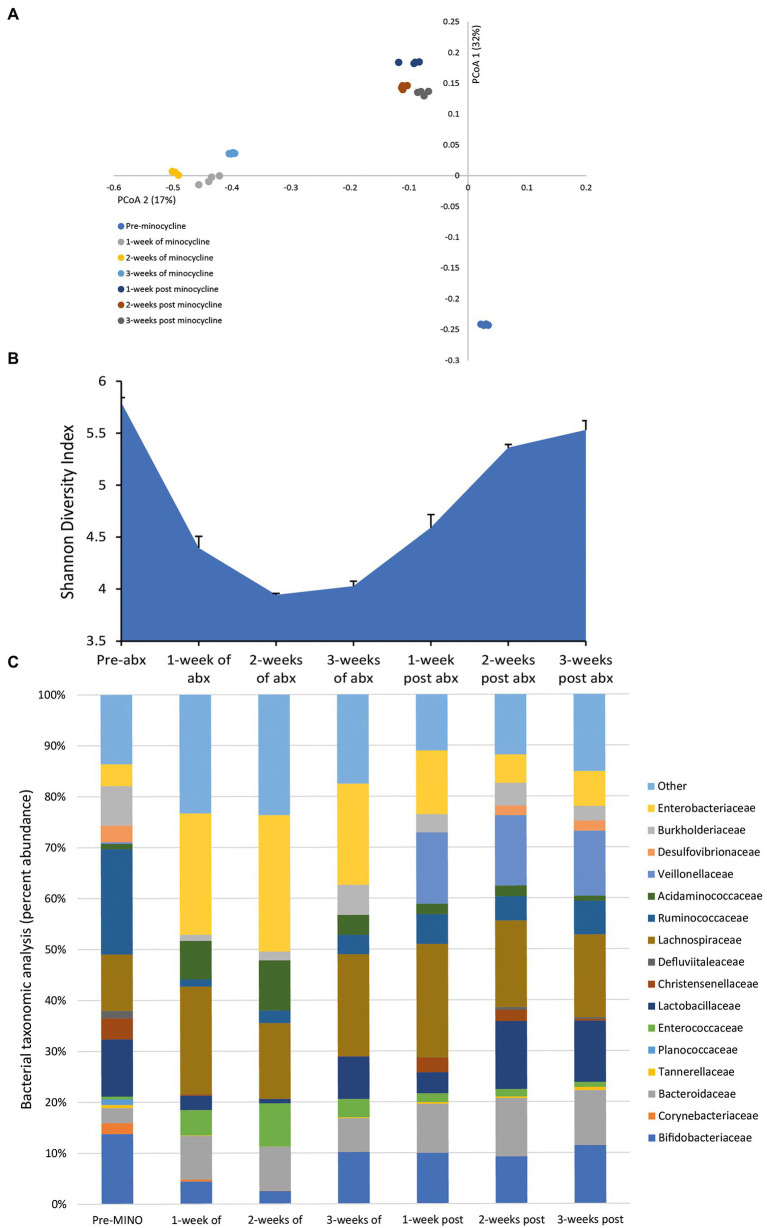
Effect of minocycline on the human microbiota. Changes to the microbiota profile during each week of minocycline instillation and the recovery phase shown as a Principal Coordinate Analysis plot **(A)**. Results shown are four technical replicates. Changes in the microbial diversity after exposure to minocycline and the recovery phase, as measured by the Shannon diversity index from four technical replicates **(B)**. Bacterial taxonomic abundance changes in response to minocycline instillation **(C)**. Stacked bar graphs are mean percent abundance, from four technical replicates, analyzed from 16S rRNA sequencing.

Administration of minocycline, once daily for 3 weeks, achieved a mean bioactive concentration of 27.4 mg/L (range over 3 weeks dosing period, 22.19–36.69 mg/L) in the gut model. This caused profound changes to the bacterial populations; the microbial populations markedly shifted from pre-minocycline levels, and we observed a major decline in the microbial diversity (Figures 3A,B). The reduced microbial diversity was slow to recover, even after antibiotic withdrawal. The minocycline-induced microbiota dysbiosis was characterized by a contraction in the abundances of Bifidobacteriaceae (↓ 10.4%) and Lactobacillaceae (↓ 10.5%), and increased abundances of Enterococcaceae (↑ 8.5%) and Enterobacteriaceae (↑ 22.4%; Figure 3C). However, the abundance of several bacterial families failed to recover to pre-antibiotic levels; Corynebacteriaceae, Planococcaceae, and Ruminococcaceae either remained undetectable or only detected at a low abundance at 3 weeks post-antibiotic.

### Effect of Doxycycline on the Microbiota

**Figure 4:**
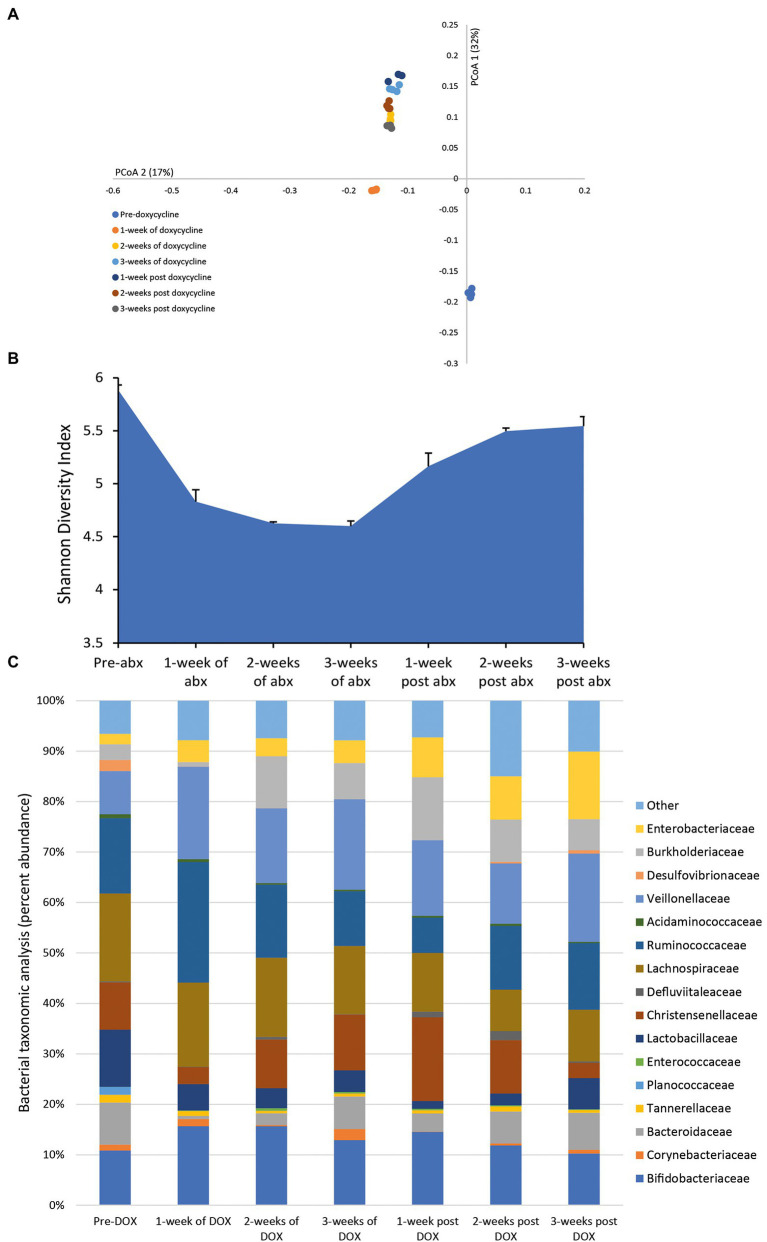
Effect of doxycycline on the human microbiota. Changes to the microbiota profile during each week of doxycycline instillation and the recovery phase shown as a Principal Coordinate Analysis plot **(A)**. Results shown are four technical replicates. Changes in the microbial diversity after exposure to doxycycline and the recovery phase, as measured by the Shannon diversity index from four technical replicates **(B)**. Bacterial taxonomic abundance changes in response to doxycycline instillation **(C)**. Stacked bar graphs are mean percent abundance, from four technical replicates, analyzed from 16S rRNA sequencing.

Following a once daily dosing of doxycycline for 3 weeks, we detected a mean bioactive concentration of 23.8 mg/L (range over 3 weeks dosing period, 17.41–28.04 mg/L) in the model. This dosing regimen of doxycycline resulted in continuous shifts in the microbiota with an increasing variation from pre-antibiotic levels and reduced microbial diversity (Figures 4A,B). Doxycycline-induced changes to the microbiota was characterized by a contraction of Lactobacillaceae (↓ 6.8%) and Bacteroidaceae (↓ 7.2%) abundancies during antibiotic instillation, whereas the abundancies of Burkholderiaceae (↑ 9.5%) and Enterobacteriaceae (↑ 6%) expanded (Figure 4C). During the recovery phase, the abundance of Lactobacillaceae, Enterobacteriaceae and Burkholderiaceae populations did not recover to pre-antibiotic levels.

### Comparative Effect of Antibiotics on the Gut Microbiota

**Figure 5:**
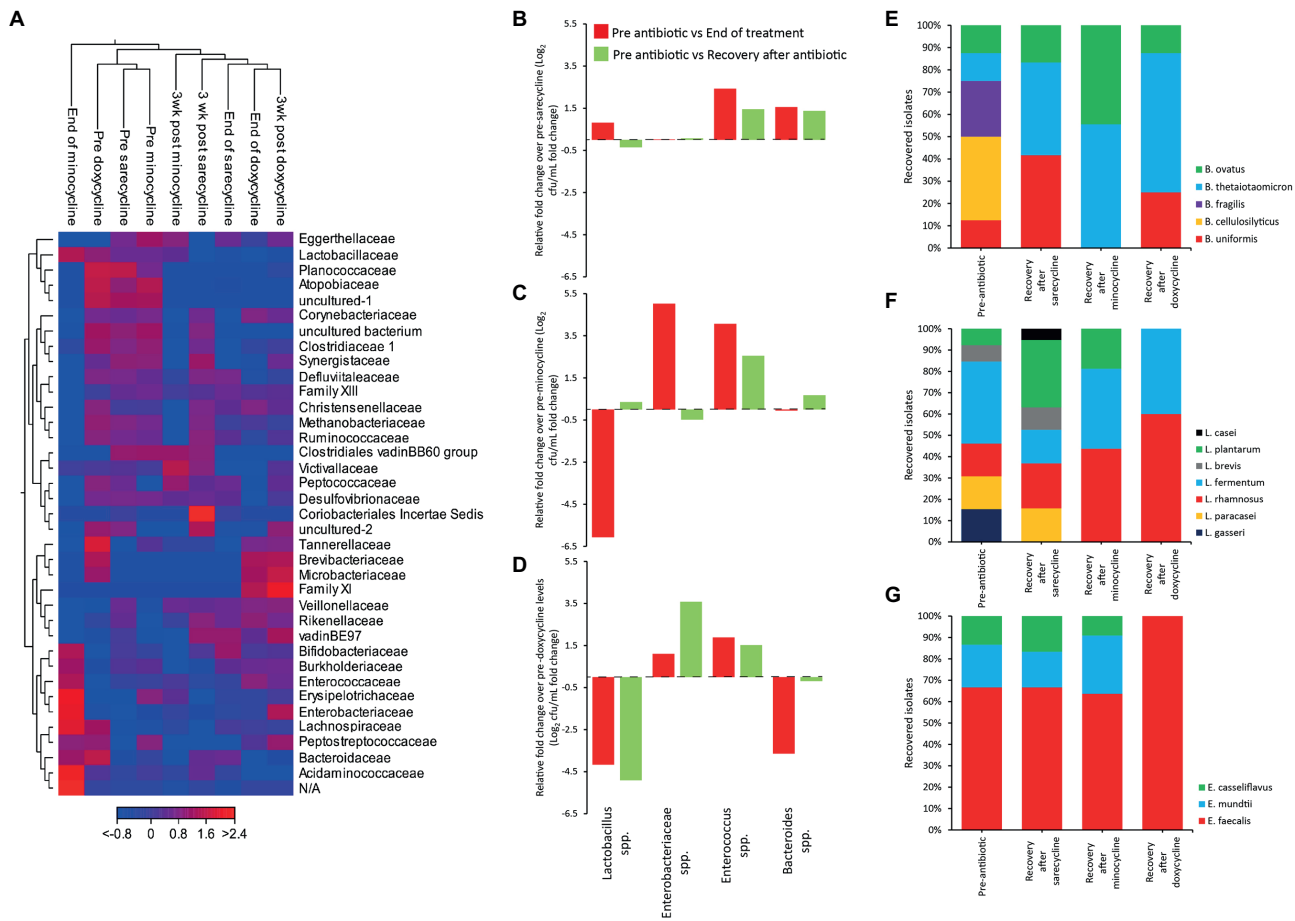
Comparative analysis of the effect of antibiotics on the human microbiota. Heat map comparing the microbial abundance before antibiotic administration, at the end of antibiotic, and at the end of the recovery phase **(A)**. Fold change in bacterial recoveries at the end of antibiotics (red bars) and at the end of recovery phase (green bars) compared with pre-antibiotic levels, for sarecycline **(B)**, minocycline **(C)**, and doxycycline **(D)**. Fold change levels calculated from microbial enumeration on selective agars shown in Supplementary Figure S2. *Bacteroides* **(E)**, *Lactobacillus* **(F)**, and *Enterococcus* **(G)** species diversity from selective agar plates before antibiotics and at the end of recovery phase. Approximately 20 colonies from each agar type, for each model, at each time point were identified by MALDI-TOF.

To explore the effects of each antibiotic on the microbiota across each independent gut model, a heat map was constructed, with clustering based on Euclidean distance, showing the changes in the relative abundances of the bacterial families over time (Figure 5A). Prior to antibiotic exposure, the three models clustered together, showing that the microbial populations in each model were highly similar. The sarecycline-induced disruption profile showed the least changes to the microbiota in comparison with minocycline and doxycycline. Microbiota recovery following sarecycline showed that most populations recovered; however, Planococcaceae and Atopobiaceae remained in low abundance. Comparatively, minocycline caused the most disruption to the microbiota, with an over representation of Enterobacteriaceae and Enterococcaceae, and the loss of Ruminococcaceae, Clostridiaceae, and Synergistaceae; all these populations failed to recover to pre-minocycline levels.

Direct enumeration of the key gut microbial populations after antibiotic therapy and during the recovery phase, compared with pre-antibiotic levels, showed that sarecycline had the lowest propensity to cause dysbiosis, with no significant change to the *Lactobacillus*, Enterobacteriaceae, and *Bacteroides* species (Figure 5B; Supplementary Figure S2). Sarecycline did induce a 2.4-fold increase in *Enterococcus* species by the end of sarecycline therapy (*p* ≤ 0.05); however, this population returned to pre-sarecycline levels during the recovery phase. In contrast, minocycline caused a 6.1-fold reduction in the *Lactobacillus* spp., and a 5- and 4.1-fold increase in Enterobacteriaceae and *Enterococcus* spp., respectively (Figure 5C; Supplementary Figure S2); however, except for *Enterococcus* spp., these microbial populations returned to pre-antibiotic levels during the recovery phase. Exposure to doxycycline caused significant (*p* < 0.05) reductions to the *Lactobacillus* (−4.2-fold) and *Bacteroides* (−3.6-fold) populations and increased levels of *Enterococcus* spp. (1.9-fold; Figure 5D; Supplementary Figure S2). Interestingly, during the recovery phase, doxycycline was associated with a 3.6-fold increase in the Enterobacteriaceae population, while *Lactobacillus* species remained significantly different to pre-antibiotic levels (*p* < 0.05).

We next compared species diversity during the recovery phase following antibiotic exposure compared with pre-antibiotic diversity. While only doxycycline negatively affected the *Bacteroides* population upon exposure, there was a change in the dominant *Bacteroides* species after exposure to all antibiotics (Figure 5E). Prior to each antibiotic, five different species of *Bacteroides* were isolated; however, all antibiotics depleted the *Bacteroides cellulosilyticus* and *Bacteroides fragilis* populations, and only *Bacteroides thetaiotaomicron* and *Bacteroides ovatus* recovered following minocycline exposure. Of the six most dominant *Lactobacillus* species identified in each model, sarecycline did not affect the *Lactobacillus* species diversity, whereas recovery following minocycline and doxycycline exposure was associated with a reduction in *Lactobacillus* diversity (Figure 5F). Similarly, the recovered diversity of the *Enterococcus* populations following sarecycline and minocycline exposure were similar, but doxycycline was associated with expansion of *Enterococcus faecalis* as the most dominant *Enterococcus* species (Figure 5G).

## Discussion

Broad-spectrum antibiotics can impact composition, diversity, and equilibrium of the gut microbiota with long-term dysbiosis effects (Dethlefsen et al., 2008; Elvers et al., 2020). Antibiotic-induced alterations of the microbial composition can last several months or years, and in some cases the microbiota does not return to the original profile, even after short-term (7 day) exposure (Dethlefsen and Relman, 2011; Francino, 2016; Elvers et al., 2020). Systemic antibiotic remains a mainstay treatment for moderate-to-severe acne vulgaris (Armstrong et al., 2020). Minocycline, doxycycline and the recently approved sarecycline are among the available and most commonly prescribed treatment antibiotics (Deeks, 2019; Moore et al., 2019; Baldwin, 2020). Treatment courses can extend for long periods, and despite antibiotic stewardship initiatives for dermatologic conditions, a retrospective analysis from 2004 to 2013 found that the mean duration for antibiotic therapy remains long. Mean antibiotic prescription durations were approximately 192 days when prescribed by dermatologists and approximately 213 days when prescribed by non-dermatologists (Barbieri et al., 2016, 2017). Replicating this duration of antibiotic exposure using *in vitro* gut models is prohibitive; however, the 3-week dosing period was sufficient to investigate the maximum drug impact on the gut microbiota, given that all antibiotics reached peak stable concentrations within 10 days of dosing.

The use of broad-spectrum antibiotics in narrow indications, such as acne vulgaris or Gram-positive cutaneous infections, can exert indiscriminate selective pressure that leads to bacterial resistance. Furthermore, clinical and experimental evidence suggests that gut dysbiosis may play a critical role in the pathogenesis of IBD (Hviid et al., 2011; Nishida et al., 2018). As such, there is a need to understand the impact that these antibiotics may have on the human microbiota.

Using three clinically reflective *in vitro* colonic models, we investigated the long-term effects of minocycline, doxycycline and sarecycline in the gut microbiota. The study used one biological replicate per testing condition; however, these models have been shown to consistently replicate the impact of antimicrobials on the human microbiota (Macfarlane et al., 1998), and the model observations correlate well with clinical trials and *in vivo* studies (Moura et al., 2019; Buckley et al., 2020; Roberts et al., 2020). Furthermore, the use of a multi-donor fecal slurry allows for a comprehensive recapture of the human microbiota (Buckley et al., 2021), which was also observed in this study (Supplementary Figure S1). As previously observed (Normington et al., 2021), prior to antibiotic dosing all models showed a similar bacterial composition, recapturing the profile of the fecal slurry used at the start of the experiment.

Unlike doxycycline and minocycline, sarecycline activity is characterized by a reduced impact in Gram-negative enteric bacterial populations such as Enterobacteriaceae (Zhanel et al., 2018). In this experiment, selective culture showed these bacterial populations remained stable throughout the experiment, while sequencing analysis revealed a brief expansion (<4%) in relative abundance of Enterobacteriaceae during antibiotic instillation. Such an effect is far less pronounced than those observed for the tetracycline-derivatives omadacycline and eravacycline in the gut model, both reporting a ~2 log_10_ cfu/ml expansion of Enterobacteriaceae (Moura et al., 2019; Buckley et al., 2020). Overall, sarecycline instillation in the gut model was not associated with overgrowth of opportunistic pathogens.

On the contrary, minocycline and doxycycline led to an expansion of either Enterococcaceae and/or Enterobacteriaceae in the gut model. Both these antibiotics caused large declines in Lactobacillaceae; minocycline also depleted Bifidobacteriaceae, while doxycycline caused a marked decline in Bacteroidaceae populations. These variations in bacterial abundance were accompanied by the decay in microbiota diversity for both antibiotics, which prevailed 3 weeks after antibiotic dosing ended. This was further evidenced by selective culture of *Bacteroides* species, showing that only *B. thetaiotaomicron* and *B. ovatus* recovered following minocycline treatment.

Similar to our gut model observations, Angelakis et al. reported a decline in *Bacteroidetes* and *Lactobacillus* populations in the gut microbiota of Q fever endocarditis patients treated with 100 mg of doxycycline twice daily for 18 months, in combination with hydroxychloroquine (Angelakis et al., 2014). In another study, a doxycycline dosage of 150 mg daily for 10 days was associated with a decline in diversity of *Bifidobacterium* populations and the increase of the tetracycline resistance gene *tetW* among *Bifidobacterium* isolates (Mättö et al., 2008). The impact of doxycycline on *Bifidobacterium* diversity and promotion of antibiotic resistance among these gut commensals could explain the stable relative abundance of Bifidobacteriaceae observed in the gut model dosed with this antibiotic.

Taken together, these results show that sarecycline exposure resulted in limited disruption to the composition of the gut microbiota, compared with minocycline and doxycycline. Following sarecycline withdrawal, the recovery of composition and diversity of the microbiota was similar to pre-sarecycline levels, whereas minocycline and doxycycline exposure had profound effects on the diversity and composition of the gut microbiota that did not fully recover when treatment was discontinued.

A healthy gut microbiota is essential in preventing opportunistic infections such as those caused by *C. difficile* or gram-negative enteric pathogens. When this balance is perturbed, particularly by broad-spectrum therapeutics such as minocycline and doxycycline, dysbiosis can persist for lengthy periods (Dethlefsen and Relman, 2011) and promote antimicrobial resistance (Moore et al., 2019). Favoring antibiotics with targeted bacterial activity such as sarecycline, can help preserve microbial homeostasis, particularly in patients with associated metabolic or inflammatory diseases impacted by microbiome health (e.g., inflammatory bowel disease; DeGruttola et al., 2016).

## Data Availability Statement

The datasets presented in this study can be found in online repositories. The names of the repository/repositories and accession number(s) can be found at: https://www.ncbi.nlm.nih.gov/, PRJNA744500.

## Ethics Statement

The collection and use of human fecal samples from healthy adult volunteers was performed under informed consent and was approved by the Leeds Institute of Health Sciences and Leeds Institute of Genetics, Health and Therapeutics and Leeds Institute of Molecular Medicine, University of Leeds Joint Ethics Committee (reference MREC 15-070—Investigation of the Interplay between Commensal Intestinal Organisms and Pathogenic Bacteria).

## Author Contributions

IM, AB, and MW designed and overviewed the experimental studies. AB, IM, WS, EC, DE, and JA conducted the experiments. IM and AB analyzed the experimental data and wrote the manuscript with additional input from MW, AG, and EF. All authors contributed to the article and approved the submitted version.

## Funding

This study was financially supported by the Almirall S.A. The funder had no involvement in experiment design and data analysis but has approved the final manuscript.

## Conflict of Interest

IM has received funding to attend conferences from Techlab, Inc. AG is the Head of R&D at Almirall (United States). EF formerly Almirall Global Medical Affairs (currently with Moderna). MW has received honoraria for consultancy work, financial support to attend meetings and research funding from Astellas, AstraZeneca, Abbott, Actelion, Alere, Bayer, bioMérieux, Cerexa, Cubist, Da Volterra, Durata, Merck, Nabriva Therapeutics plc, Pfizer, Qiagen, Roche, Seres Therapeutics Inc., Synthetic Biologics, Summit, and The Medicines Company. AB has received financial support to attend meetings and research funding from Seres Therapeutics Inc., Motif Biosciences plc., Nabriva Therapeutics plc, Tetraphase Pharmaceuticals, Almirall SA, GlaxoSmithKline plc, and Hayashibara Co. Ltd.

The remaining authors declare that the research was conducted in the absence of any commercial or financial relationships that could be construed as a potential conflict of interest.

## Publisher’s Note

All claims expressed in this article are solely those of the authors and do not necessarily represent those of their affiliated organizations, or those of the publisher, the editors and the reviewers. Any product that may be evaluated in this article, or claim that may be made by its manufacturer, is not guaranteed or endorsed by the publisher.
