# Supplementary material for: Profiling the Effects of Systemic Antibiotics for Acne, Including the Narrow-Spectrum Antibiotic Sarecycline, on the Human Gut Microbiota
Source: Front Microbiol. 2022 May 31;13:901911. doi: 10.3389/fmicb.2022.901911 (PMC9194605; doi:10.3389/fmicb.2022.901911)
Supplement: Supplementary file 1 [file Data_Sheet_1.PDF]

Profiling the effects of systemic antibiotics for acne, including the narrow-spectrum antibiotic sarecycline, on the human gut microbiota

Supplementary results

Figure S1. Recapturing the human microbiota in the *in vitro* gut models. Taxonomic analysis of the pooled faecal slurry and samples from the pre-antibiotic exposure period (experimental day 14) in the three independent models (S, M and D). Principal coordinate analysis (A) and mean bacterial family abundance (%) (B) based on 16S rRNA sequencing from four technical replicates for each sample.

Figure S2. Recoveries of bacterial populations from vessel 3 of model S, model M, and model D. The bacterial populations enumerated were, total obligate anaerobic bacteria (black lines), lactose-fermenting Enterobacteriaceae (red lines), *Enterococcus* spp. (green lines), and *Lactobacillus* spp. (blue lines). Results expressed as mean colony-forming units ± SD of three technical replicates. Horizontal green arrow represents the period of antibiotic dosing to all gut models.

Figure S1

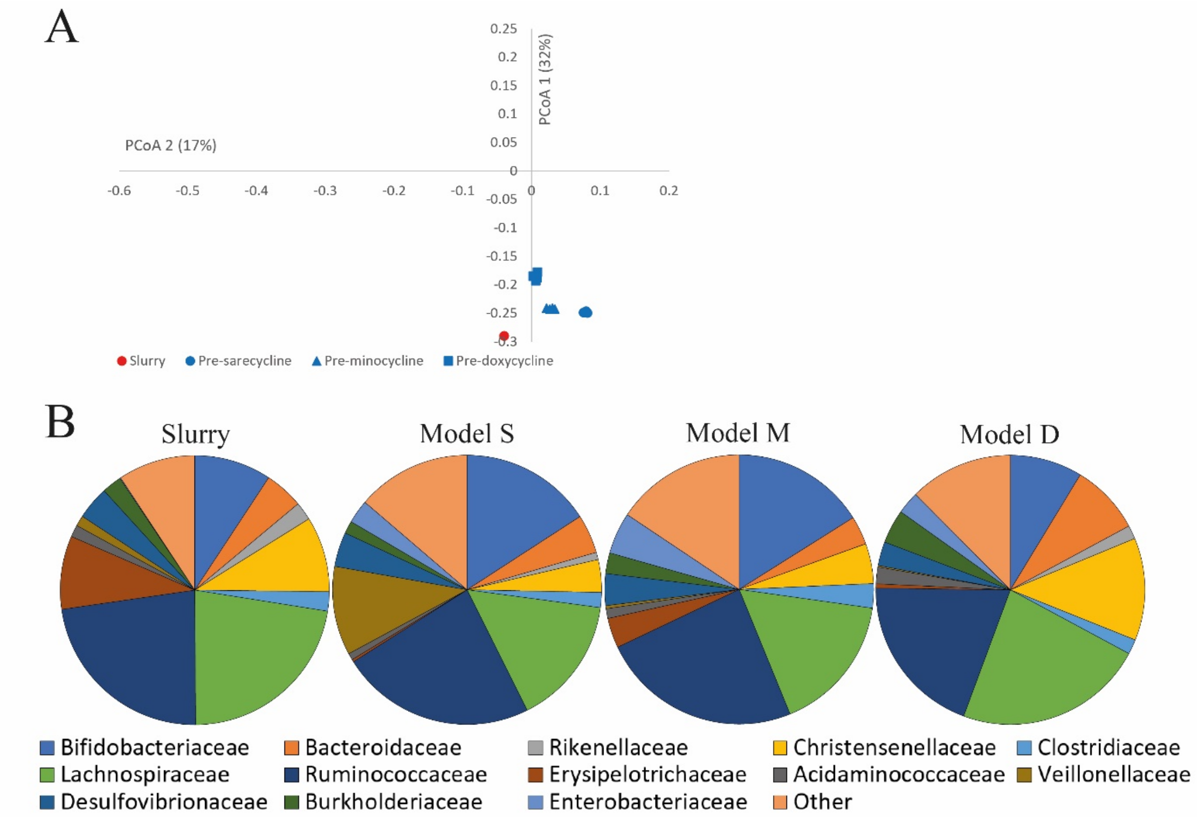
A
PCoA 2 (17%)
PCoA 1 (32%)
0.25
0.2
0.15
0.1
0.05
0
-0.05
-0.1
-0.15
-0.2
-0.25
-0.3
-0.6
-0.5
-0.4
-0.3
-0.2
-0.1
0
0.1
0.2
Slurry
Pre-sarecycline
Pre-minocycline
Pre-doxycycline
B
Slurry
Model S
Model M
Model D
Bifidobacteriaceae
Bacteroidaceae
Rikenellaceae
Christensenellaceae
Clostridiaceae
Lachnospiraceae
Ruminococcaceae
Erysipelotrichaceae
Acidaminococcaceae
Veillonellaceae
Desulfovibrionaceae
Burkholderiaceae
Enterobacteriaceae
Other

Figure S2

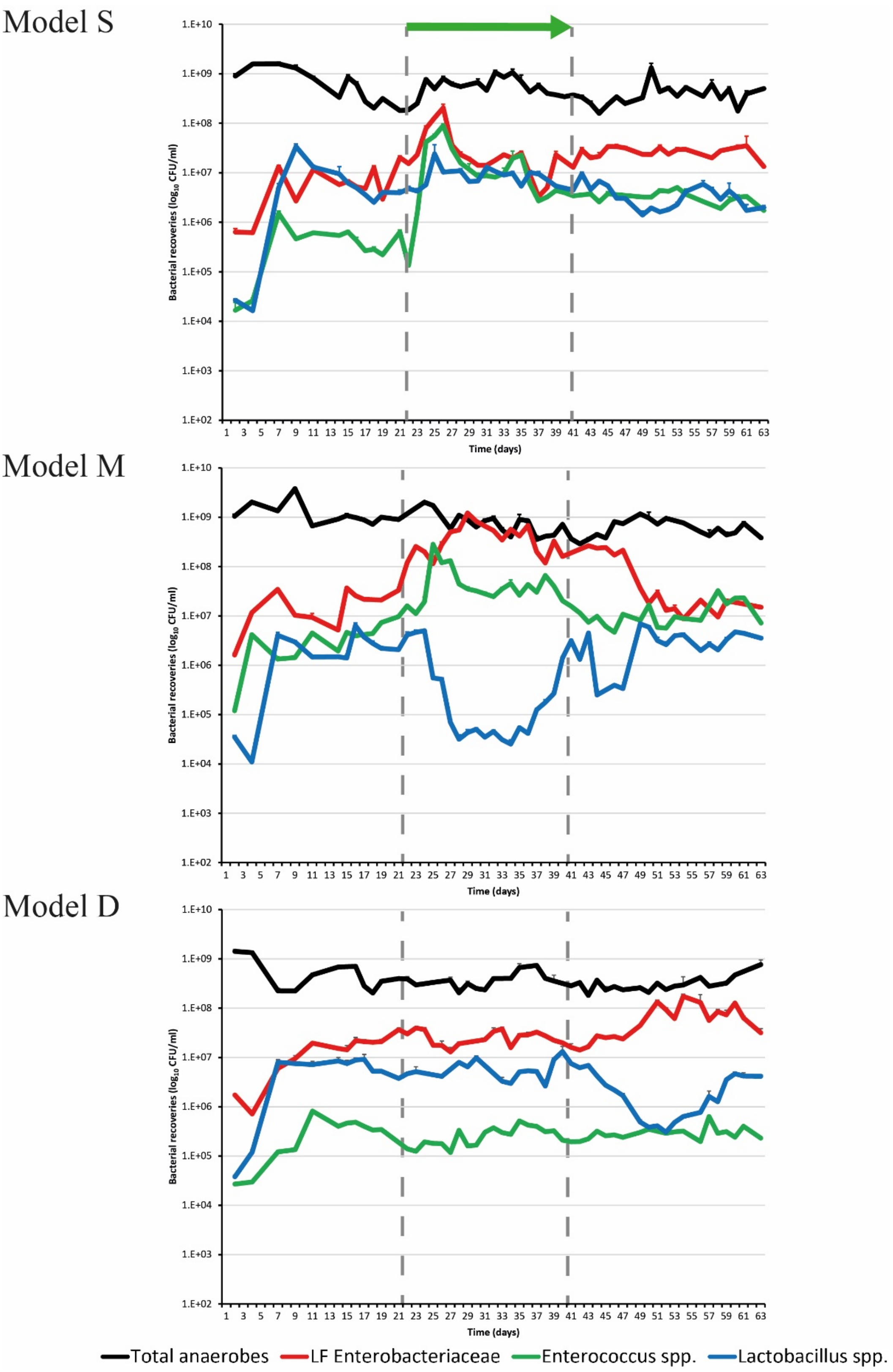
Model S
Model M
Model D
Bacterial recoveries (log10 CFU/ml)
Time (days)
1.E+10
1.E+09
1.E+08
1.E+07
1.E+06
1.E+05
1.E+04
1.E+03
1.E+02
Total anaerobes
LF Enterobacteriaceae
Enterococcus spp.
Lactobacillus spp.
